# Guiding Policymakers Toward Better AI Ethics Integration in Healthcare Regulation—Lessons from Singapore

**DOI:** 10.3390/jcm15103576

**Published:** 2026-05-07

**Authors:** Alexa Nord-Bronzyk, Bryson Ng, Tianxiang Lan, G. Owen Schaefer, Shizuko Takahashi

**Affiliations:** 1 Centre for Biomedical Ethics, Yong Loo Lin School of Medicine, National University of Singapore, Singapore 117597, Singapore; s220192@e.ntu.edu.sg (B.N.); tx.lan@nus.edu.sg (T.L.); owen_schaefer@nus.edu.sg (G.O.S.); shizuko@nus.edu.sg (S.T.); 2 Department of Biomedical Ethics, Graduate School of Medicine, University of Tokyo, Tokyo 113-0033, Japan; 3 Department of Bioethics & Humanities, University of Washington School of Medicine, Washington, DC 98195-7120, USA

**Keywords:** AI ethics, policy, gap analysis, healthcare

## Abstract

In terms of rollout, comprehensiveness, and strategy, Singapore’s regulatory landscape governing the ethical use of Artificial Intelligence (AI) in healthcare has generally kept pace with other global leaders in AI advancement. However, establishing a robust and holistic regulatory framework that evolves along with emerging technologies is not easy—especially in healthcare, where the stakes are high and resources may be limited. We conducted a structured scoping analysis of key AI regulatory and professional documents in Singapore, selected using predefined inclusion criteria. Documents were systematically mapped against Savulescu et al.’s nine categories of ethical risk, followed by cross-document comparison to identify integration gaps and inconsistencies, and benchmarking against international AI governance frameworks. These recommendations are generalizable beyond Singapore for developers, implementers, healthcare professionals and patients and include dealing with bias in AI, enhancing human productivity without deskilling, facilitating more informed decision-making, and cultivating greater knowledge exchange between clinicians and patients, to name a few.

## 1. Introduction

As the Artificial Intelligence (AI) regulatory landscape in healthcare continues to rapidly evolve, certain ethical concerns remain constant, and regulation often struggles to keep up. In terms of rollout, comprehensiveness, and strategy, Singapore’s regulatory landscape governing the ethical use of AI in healthcare has generally kept pace with other global leaders in AI advancement. However, establishing a robust and holistic regulatory framework that evolves along with emerging technologies is not easy—especially in healthcare, where the stakes are high and resources may be limited. This paper examines how Singapore’s regulatory framework addresses ethical concerns surrounding the use of AI in healthcare. It investigates how well current policies mitigate key risks identified in existing AI ethics literature and proposes improvements for local and global relevance.

We recognize that AI is not a singular, bounded entity, but a collection of technologies with varying uses, and will defer to the Nuffield Foundation’s definition of artificial intelligence as “any technology that performs tasks that might be considered intelligent—while recognizing that our beliefs about what counts as intelligent may change over time” [1]. We will also only be referring to clinical AI tools and their practical applications, though some arguments could also apply to AI supporting administration and population health management.

Regulatory frameworks may have varying roles, including risk mitigation, enhancing AI deployment, or attracting investment through a comparatively well-defined framework. This paper will explore the role of risk mitigation by endorsing Savulescu et al.’s review of the nine main risks arising from the use of AI in medicine [2] That is not to say that other roles of regulation are unimportant or not factored into the motivations of Singapore’s governance framework, but that exploring those roles is outside of the scope of this paper. Additionally, we endorse Savulescu et al.’s list of risks as it is consistent with the literature and comprehensive in scope. These risks are outlined in Table 1 below:

These concerns reflect a comprehensive understanding of the risks posed by inappropriate or unethical development, implementation, or use of AI in healthcare. By identifying potential issues arising from the above risks, ethical AI regulations can more effectively employ risk mitigation strategies.However, if regulations and ethics mis-align, crucial gaps may emerge where relevant risks are not adequately addressed or mitigated.

In order to provide a critical assessment of the extent to which existing approaches in Singapore align with the risk-based ethical framework outlined above, we conducted a structured scoping commentary. Our aim is normative and analytical, following the view advanced by Schiff Reet al. that mapping consensus and dissensus—and scrutinizing where progress is needed—is itself a legitimate and rigorous mode of inquiry in AI ethics research [3]. We adopted this approach rather than a comprehensive systematic review or exhaustive gap analysis because, while existing documentation is relevant, our work is substantially driven by normative analysis. Furthermore, the Singaporean AI ecosystem is relatively well-contained and so we were able to effectively identify the key/central documents through searching for the ethical codes on the websites of the Health Sciences Authority, Ministry of Health, Singapore Medical Council, Agency for Care Effectiveness, Allied Health Professions Council, Singapore Pharmacy Council, Singapore Nursing Board, and Singapore Dental Council.

Like many countries, Singapore currently has a patchwork of regulatory and guidance documents regarding AI in healthcare that may be comprehensive in isolation but struggle to integrate with one another and the wider AI landscape [4,5]. At the time of writing this paper, the main documents in Singapore included (1) HSA Regulatory Guidelines for Software Medical Devices—A Life Cycle Approach, (2) MOH Artificial Intelligence in Healthcare Guidelines (AIHGle), and (3) Regulatory Guidelines for Telehealth Products: Medical Health Branch. Generative AI raises a set of unique ethical considerations that are too expansive for the scope of this paper, and thus we did not review the Model AI Governance Framework by Singapore’s Infocomm Media Development Authority and AI Verify. In order to keep the analysis broad enough to sufficiently illuminate legitimate integration gaps, we reviewed the main documents in Singapore that directly address AI in health by focusing on (1) hard and soft regulation, (2) ethical codes, and (3) bridging documents (ACE overview). This strategy aims for functional representativeness rather than completeness.

Documents were selected for inclusion if they met three criteria: (1) issued by a Singapore government body, statutory board, or professional council; (2) directly governing or guiding the use of AI in healthcare, regulating software medical devices, or establishing the professional ethical conduct of clinicians who interact with AI tools; and (3) publicly accessible at the time of writing. Documents were excluded if they addressed only generative AI governance in non-clinical contexts—hence the exclusion of the IMDA Model AI Governance Framework and AI Verify—or if their relevance to clinical AI use was only incidental. This yielded nine documents organized into two groups: AI-specific regulatory and guidance documents (n = 4), and professional codes of ethical conduct (n = 5) (see Appendix C). The separation was deliberate: our central analytical question was whether the emerging regulatory and guidance architecture for AI aligns with the ethical standards already binding on healthcare professionals, and where misalignment creates governance gaps.

While the documents analyzed provide a snapshot of the landscape of regulatory and guidance documents in Singapore, there are many relating and complementary documents that would contribute to the ethical evaluation of AI in healthcare such as privacy and data concerns in the Personal Data Protection Act (PDPA), Advisory Guidelines on use of Personal Data in AI Recommendation and Decision systems, and Model AI Governance Framework for Generative AI, among others. Generative AI in particular raises a set of unique ethical considerations that are not addressed in this report as the selected list of documents does not include generative AI-specific guidance. However, due to time constraints, the documents selected are sufficient to understand the major areas of ethical considerations in the ethical codes in Singapore so that a structured scoping commentary could produce meaningful results and illuminate possible integration gaps. Future work would aim to include the full landscape of documents.

Analysis proceeded in three stages. First, each document was mapped against Savulescu et al.’s nine risk categories to identify which risks each document explicitly addresses, partially addresses, or omits. Second, cross-document comparison surfaced integration gaps—ethical concerns that appear in some documents but not others, or that are addressed inconsistently in ways that could generate uncertainty for developers, implementers, or clinicians in practice (see Appendix B). Savulescu et al. was chosen as the organizing framework rather than alternatives because it was developed with Singapore’s context in mind and is already influential in regional bioethics discourse. Third, Singapore’s framework was benchmarked against key international AI governance frameworks to assess alignment with global standards and identify further areas for improvement (see Appendix D) These include the US-based Food and Drug Administration (FDA)’s 2021 Action Plan [6]; the US AI Bill of Rights (2022) [7]; the UK’s Medicines and Healthcare products Regulatory Agency (MHRA) 2024 roadmap [8]; the European Union’s AI Act (2024) [9]; Australia’s Safe and Responsible AI in Healthcare Legislation and Regulation Review (2024) [10]; guidelines under China’s National Medical Products Administration [11]; Brazil’s recent AI Bill (2024) that operates on a risk-based regulatory model [12]; and documents that inform Japan’s regulatory landscape, including the Pharmaceuticals and Medical Devices Act, Digital Transformation Action Strategies in Healthcare for Software as a Medical Device (SaMD), and the Next Generation Medical Infrastructure Act. Recommendations were then derived from the gaps identified across all three stages and organized by stakeholder group [13,14,15]. Rather than a comprehensive systematic review or exhaustive gap analysis, this constitutes a structured scoping commentary: its aim is normative and analytical rather than epidemiological, following the view advanced by Schiff et al. that mapping consensus and dissensus—and scrutinizing where progress is needed—is itself a legitimate and rigorous mode of inquiry in AI ethics research [3].

Our recommendations are intended to guide governments and policymakers in developing future AI ethics guidelines for healthcare in Singapore and internationally by provoking conceptual analysis surrounding such issues We note that one limitation of our paper is moving quickly from conceptual analysis to regulatory prescription. Due to time constrains we would like to highlight that our recommendations are not strong prescriptions and serve as motivators for thinking about how to shape policy. We hope future work will address this limitation by demonstrating why our recommendations are appropriate for Singapore’s institutional setting. While our analysis is Singapore-specific, the recommendations are not necessarily rooted in local, cultural, legal, or political contexts and, as such, can be more broadly deployed. Therefore, what we propose here is not for other countries to consider our recommendations as necessarily relevant for their own, but to offer a process for identifying and understanding their own possible gaps.

Our analysis offers a novel contribution in several ways. First, by evaluating the selected documents from the perspective of integration, we illuminate inconsistencies, ambiguities, and gaps that would not otherwise be identifiable if analyzed in isolation. Second, mapping the regulatory landscape in Singapore against the nine risks raised by Savulescu et al., our method is unique and maintains relevance to the context while providing a distinct tool that other nations may replicate for their own contexts. In particular, our method may be especially useful in jurisdictions where there may be less centralized regulatory landscapes, or that lack as robust a regulatory coordination as Singapore. In low-resource settings, for example, it may be difficult to operationalize our recommendations when there are competing priorities, making the strength of our suggestions lie in the process of identification rather than generalisability.

## 2. Analysis

An issue facing the development of AI regulation and guidance in healthcare is how new guidance and regulations will integrate with other existing policy documents in healthcare, especially where AI was previously not of concern (i.e., the ethical codes of conduct). As AI technologies progress, we must consider their compatibility with existing documents that affect the ethical considerations overlapping among them. This section will describe the process of our analysis based on the ethical risk framework of Savulescu et al., and how we translated our analysis into recommendations for how to think about filling such gaps and mitigating such risks. As mentioned, we recognize that other countries may have different gaps and risks, and thus their recommendations may look different, but the process for conceptual analysis may be generalizable for countries in similar positions.

Our analysis began with a review of four regulatory and guidance documents, along with five ethical codes of conduct in Singapore, focusing on identifying gaps in how these documents relate to one another, which could result in allowing for the risks mentioned by Savulescu et al. (see Appendix B and Appendix C). The selection of documents is meant to motivate an analysis of how the emerging regulatory and guidance documents for AI in healthcare integrate with the ethical codes. The underlying question asks whether the guidance and regulatory documents align with the ethical codes, and if not, how can these gaps be managed?

Documents were separated into two groups: (1) those pertaining to AI and (2) the ethical codes of conduct. This separation allowed us to organize our analysis based on the aims of each group, as well as identify the overlapping ethical themes and gaps to provide a synthesized analysis with pointed recommendations (see Appendix A). The analysis demonstrates areas for improvement that include both updates to current documents as well as suggestions for the addition of new documents to provide a holistic picture of what is needed for the prospect of a “Healthcare AI Ethics” in Singapore.

To further substantiate our analysis, we performed a high-level overview of international AI regulatory frameworks to see where Singapore’s regulations and guidelines sit against global standards and highlight areas for improvement (see Appendix D). By comparing Singapore’s governance framework with that of other countries, we were able to identify further gaps, which additionally substantiated our recommendations. Finally, we surveyed the literature pertaining to the gaps we identified to further develop and finalize our recommendations.

## 3. Recommendations

Based on our analysis, we suggest the following recommendations for thinking about how to mitigate the risks raised by Savulescu et al. in Singapore’s context. While each recommendation may not be entirely new, our synthesized list provides guidance for advancing AI ethics in Singapore and elsewhere that can be employed by stakeholders and policymakers in this space (See Appendix E for recommendations distinguished by relevant characteristics.).

### 3.1. Involve Patient Perspectives in the Development and Implementation of AI in Health

While AIHGle recommends involving all stakeholders in the development and implementation of AI in healthcare, it does not explicitly specify who represents patient perspectives or how such perspectives should be incorporated. This omission is notable given its emphasis on “patient-centric” design.

Three distinct questions must be addressed. First, who counts as a relevant patient voice? Reliance on convenience sampling from advocacy communities risks systematically over-representing patients who are health-literate, digitally fluent, and already embedded within formal healthcare systems. This bias is ethically significant, particularly for AI tools deployed in context-sensitive domains such as mental health, chronic disease management, fertility care, and end-of-life care, where vulnerable populations may be disproportionately affected. Guidelines should therefore require that engagement strategies actively recruit across differences in age, language, health literacy, disability status, and condition severity. Developers and implementers should also be required to document how they have identified and addressed failures of representativeness.

Second, at what stage does patient input carry weight? Engagement at the post-deployment feedback stage, the modal form in current practice, cannot correct for value assumptions embedded in training data, outcome metrics, or risk-benefit thresholds set during design. If patient-centricity is to be substantive, guidelines should distinguish between engagement at the design stage (where input can shape what the tool optimizes for), the validation stage (where patient-defined outcomes can supplement clinical metrics), and the post-deployment stage (where structured feedback loops can trigger review). Each stage requires different recruitment strategies, competencies, and institutional commitments.

Third, and most critically, what decision rights does patient input carry? Is it advisory, or does it carry co-design authority that can trigger mandatory review or delay procurement approval? Guidelines should answer each question explicitly. These questions also connect to the paper’s justifiability argument: an AI tool cannot be justified to patients if the criteria of justification were set without them.

Two additional challenges warrant consideration. First, we lack sufficient empirical evidence about what patients across conditions, demographics, and treatment stages actually want from AI involvement in their care [16]. Existing literature on patient perspectives remains limited in scope and geographically concentrated, meaning that recommendations for engagement processes risk encoding assumptions that are themselves untested. Future guidelines should explicitly call for investment in qualitative, deliberative, and longitudinal research conducted with diverse patient populations at each stage of the development and procurement cycle, with findings feeding iteratively into regulatory revision.

Second, cultural context shapes how people evaluate AI involvement, and guidelines should not assume uniform patient preferences across populations. Cross-cultural empirical work on lay attitudes toward AI, for example, comparisons across the US, UK, China, and Singapore, demonstrates that moral evaluations of AI vary significantly by national context [17]. Although healthcare-specific data remain limited, these findings suggest that a uniform model of patient engagement is inappropriate. In contexts where empirical evidence is limited and cultural variability is known to matter, a stance of regulatory humility is warranted. Guidelines should therefore require culturally sensitive needs assessments as part of the validation process, following the precedent of systematically mapping cultural variation in ethical intuitions as design input. Practical infrastructure, such as Singapore’s REACH (Reaching Everyone for Active Citizenry @ Home) platform and deliberative engagement panels, offer promising mechanisms for incorporating diverse public input. However, these must be supported by regulatory requirements specifying how disagreements between patient and developer priorities are to be documented and adjudicated.

### 3.2. Indicate That AI-Based Tools May Be Evaluated Based on Their Justifiability If They Are Not Explainable

Explainability refers to the ability of AI systems to provide adequate reasons or explanations for their decisions, output, or recommendations. For Artificial Intelligence-based Decision Support Systems (AI-DSS) in particular, explainability describes “the ability of either the AI-DSS to give explanations for its decision-making or an agent to explain the decision-making of an AI-DSS to a certain degree” [18]. The explainability of a tool is often touted as an important AI ethics principle to mitigate risk without sufficient discussion of what is at the crux of importance about explainability [19,20,21]. Explainability may not directly indicate how risky a tool is; nevertheless, the extent to which end-users understand and feel comfortable using it will affect both its proper use and overall success [22].

Despite the push for explainability in emerging AI tools, there are various examples in healthcare where devices or medicines may be used or prescribed by healthcare professionals who do not fully understand how or why they are successful (e.g., aspirin) [23]. Thus, the explainability of AI tools needn’t always be a prerequisite for patients’ trust in the healthcare system or their informed decision. For AI tools whose inner logic is not transparent, justifiability may be more ethically meaningful for evaluating those tools. Justifiability aims to ensure that a tool’s use leads to beneficial outcomes. In healthcare, it means professionals can justify their reliance on AI outputs, while also ensuring these decisions align with patient values and preferences [24].

However, explainability and justifiability are indeed contested concepts, and we recognize that their substitutability should be dealt with scrutiny. For example, explainability in research reaches beyond trust and patient comfort to support other functions such as auditability, contestability, error analysis, and legal accountability. Therefore, we would like to emphasize that our above argument is contextualized around trust and the patient experience and may be contested in other contexts. We also note that explainability may be more important in the use of higher-risk diagnostic tools such as PCR tests, where immediate, critical interventions may be needed.

### 3.3. Developers and Implementers Should, Depending on the Context of Use, Warn Users Against the Tendency to Anthropomorphize AI-Based Tools

Trust is a term generally applied to moral agents and not tools. Attributing trust toward AI risks anthropomorphizing AI tools (e.g., imbuing AIs with mental capacities uniquely associated with humans, such as emotional capacity or moral agency) [25,26,27]. “Trust in AI” is best understood metaphorically, and it is the developers, implementers, and healthcare professionals who should be trusted to deploy and use AI safely and ethically. We recommend emphasizing reliability instead, as this focuses on the tool’s clinical effectiveness—what truly matters ethically is whether its outputs are valid and useful [24]. Nonetheless, we recognize the institutional and sociotechnical nature of how trust operates and do not wish to claim there is no place for trust in the AI ethics ecosystem. Instead, we wish to caution against the anthropomorphizing of AI-based tools when the outcome may result in risks to the user (i.e., psychological harm).

### 3.4. Provide Guiding Tools for Ethical Analysis of Proportionality in Data Collection and Making Ethical Trade-Offs in General

AI ethics does not provide a one-size-fits-all ethical theory or a certain algorithm to come to final conclusions. Often, principles will conflict, and difficult choices will need to be made in evaluating the most ethically appropriate choice. For example, when utility and equity are at odds, which principle should be upheld [28]? Proportionality in AI ethics seeks to address the tensions between different ethical principles by carefully weighing the risks and benefits to reach an appropriate resolution. Applying proportionality can help implementers and developers navigate real-world situations and make necessary ethical trade-offs [29,30] (see Appendix F for case study).

### 3.5. Publish Approved AI/ML-Enabled Devices for Medical Use

Although Singapore does publish a listing of registered medical devices in the existing Singapore Medical Device Register (SMDR) under the Health Sciences Authority, it lacks a dedicated list of approved AI and ML medical devices [31]. A publicly available list of approved AI/ML-enabled healthcare devices, similar to the one provided by the US Food and Drug Administration, helps health professionals clearly see which AI tools are approved, thereby promoting greater trust in government oversight of these technologies [32] (Note that it appears Singapore does provide such a list, but it is not publicly available. One must obtain a Corpass account which requires a business affiliation). Making such a list available also ensures devices meet strict safety and efficacy standards, which helps protect patients and reduce risks. Additionally, it encourages innovation by offering clear regulatory pathways for developers and guidance for new entrants in the AI/ML field. Such a move would be operationally feasible by requiring minimal regulatory burden, low cost, and high impact.

### 3.6. Extend Personal Data Protection Beyond Identified Data

A key challenge posed by the advent of AI is that it can be used to reveal the partial or full identity of the person from certain deidentified medical data [33]. This can result in group-level discrimination when AI-based tools identify patients’ social groups and/or race, such as through radiological images where outwardly visible bodily features are not depicted [34]. Other AI models were found to widely vary in the accuracy of automated chest X-ray diagnosis across racial and other demographic groups despite only having access to the chest X-ray itself [35]. This may open doors to privacy breaches and even malicious use of data, such as covert surveillance and discrimination.

It is plausible that the data privacy requirements for healthcare providers follow the regulations and guidelines related to PDPA. However, according to Part 3 of the Advisory Guidelines on the PDPA for Selected Topics, the Data Protection Provisions in the PDPA do not apply to the third-party recipient if only de-identified data are shared with the latter. The guideline considers the possibility of re-identification, but only advises the sharing organization to use “contractual safeguards” to prevent this from happening. Given the above challenges posed by AI, relevant authorities may consider instituting mandatory technical safeguards for the sharing of medical data by public or private health institutions, rather than those currently afforded by the PDPA. This should include expanding the range of scenarios when the Data Protection Provisions apply beyond de-identified data, and instituting statutory amendments that strictly regulate the use of AI for surveillance.

### 3.7. Clarify the Distribution of Liability Between Healthcare Professionals, Implementers and Developers

There needs to be a clearer distribution of responsibility when AI systems fail. This involves defining the legal and ethical liability shared by healthcare professionals and developers or service providers when patients are harmed. Additionally, this should be extended to other sectors, taking into account the perspectives of relevant stakeholders’ views [36]. This can be especially complicated due to fluctuations of involvement among said parties, so a standardized approach should be avoided to address the complexities of each case [3]. Plausible frameworks ought to consider the different types of liability pertinent to the deployment and use of AI systems in the healthcare setting, including those that are specific to healthcare professionals (e.g., user liability and its relationship with professional negligence), implementers (e.g., institutional and procurement liability), and developers (e.g., developer liability) (for more on these different types of liability, see [37]). Approaches should be tailored to the level of human oversight necessary in using the tool, the responsibility structure of those involved in its success, and the level of risk the tool poses in terms of harm and magnitude. Doing so would help ensure that accountability is both appropriately assigned and practically enforceable via regulatory efforts that recognize the multifaceted concept of AI liability. Approaches should be tailored to the level of human oversight necessary in using the tool, the responsibility structure of those involved in its success, and the level of risk the tool poses in terms of harm and magnitude.

Chan’s analysis of Singapore’s existing laws provides a good starting point for clarifying legal liability in medical AI use. For doctor/hospital fault, the key idea in Singapore medical negligence (for diagnosis and treatment) is that a doctor will generally not be deemed negligent if they acted in accordance with a practice accepted by a responsible body of medical opinion, provided that view is logically defensible; ultimately, the question is whether the doctor acted reasonably, assessed based on what was known at the material time (without hindsight). For a developer/designer fault, Chan suggests negligence can arise where those parties fail to take reasonable steps to modify algorithms to prevent anticipated harms; conversely, they may be absolved where the system’s learning is unpredictable, such that there is a lack of foreseeability of risks. Their liability also depends on whether the developer/designer knows or ought to know the context of use. Chan also points out an area of legal uncertainty with respect to medical AI, that courts may need to define the relevant standard of care for medical AI itself, which can be a quickly “moving target” as technology evolves. On this view, future regulatory clarification should directly address this moving-target problem by giving clearer guidance on expected assurance/validation processes and acceptable reliance/oversight as AI performance and clinical adoption continue to develop [38]. This is especially true in the case of adaptive models that raise challenges for post-market surveillance.

Furthermore, there may be situations where professionals must dismiss AI recommendations—and could be held liable if they do not—as well as situations where they may choose to disregard AI without liability. In some cases, requiring documentation when professionals override AI recommendations may unintentionally encourage over-reliance on AI.

### 3.8. Clarify How Implementers Should Deliberate the Appropriate Amount of Human Oversight of AI Appropriate by Weighing Risks and Benefits of Full Automation

Different tools require different levels of human oversight. Maintaining a nontrivial degree of human oversight—the ‘human in the loop’ approach in AI—is widely regarded as good ethical practice in many circles. However, as technology evolves and users gain confidence in using these tools, it is important to assess when greater or lesser degrees of automation are appropriate.

It may not always be true that the addition of an AI tool will enhance the performance of a task or improve a workflow. While AIHGle does well to promote understanding of a tool’s baseline performance when measuring its clinical effectiveness, it lacks guidance on when full automation may or may not be appropriate. Stakeholders should carefully consider increasing automation only when the potential benefits clearly outweigh the associated risks [39].

### 3.9. Require Calibration of Tools to Be Culturally Sensitive

Singapore’s cultural diversity should be reflected in AI regulations by accommodating different patient values. Just as doctors must be culturally sensitive to respect their patients, AI tools should be calibrated to account for the cultural nuances of the populations they serve. Tools such as personalized patient preference predictors are a great example of where the field is heading in this regard [40].

### 3.10. Clarify When and How, If Ever, Professionals May Still Use a Biased AI

Since completely eliminating bias in AI-based healthcare tools is not feasible, it is important to clarify under what circumstances, if any, developers and implementers may market tools that show discernible bias toward certain groups. Likewise, guidance is needed on when, if ever, healthcare professionals should rely on such tools for medical decisions. For example, Muyskens et al. argue that “it is permissible to implement an AI tool with residual bias where (1) its introduction reduces the influence of biases (even if overall inequality is worsened), and/or (2) where the utility gained is significant enough and shared across groups (even if unevenly)” [28].

### 3.11. Clarify the Scope of Patient Rights in Relation to the Use of AI-Based Tools

Regulations ought to account for how patient rights regarding the use of AI in their care ought to evolve with the emergence of new AI-based tools and technologies. For instance, AI-based tools may have profound impacts on the concept of privacy for patients in the healthcare setting, given the handling of vast amounts of personal health data under such tools [41]. Whether such a right exists will vary by context. For example, if patients reveal the kind of information to AI that healthcare professionals are typically required to report to third parties (e.g., suicidal intention), should the tools be programmed to disclose such information to next-of-kin or authorities, or should an “AI confidant” observe strict confidentiality [42]?

These considerations might be handled with further regulations, as is being done in the EU with a proposal for the EU Charter for Digital Patients’ Rights. The proposal suggests patients should have the right not to be subject to automated medical decision-making and the right to meaningful human contact.

### 3.12. Guiding Documents Regarding the Doctor–Patient Relationship on Their Rights and/or Duties with Respect to AI Use in Healthcare

While there is no explicit mention of the ethical use of AI in any current codes for medical professionals in Singapore, we argue that it is essential to incorporate this aspect with careful attention to patient perspectives. Here we refer to the following codes: The Singapore Medical Council (SMC) Ethical Code and Ethical Guidelines (ECEG), SDC Ethical Code & Ethical Guidelines, The Allied Health Professions Council Code of Conduct, The Singapore Pharmacy Council’s Code of Ethics, and the Code for Nurses and Midwives. Highlighting the ethical implications of AI in healthcare is crucial for ensuring that patient rights and well-being are prioritized.

As the concerns of healthcare professionals and patients overlap, each group ought to be aware of the other’s roles and responsibilities, guiding both groups on awareness of the symbiotic relationship of their rights, roles, and responsibilities. For example, the US AI Bill of Rights recommends protecting patients from unsafe or discriminatory automated systems, safeguarding privacy, ensuring transparency about AI use, and providing patients with the option to opt out when appropriate [43]. Elsewhere, ethicists have suggested that clinicians may have an ethical obligation to disclose the use of AI systems to their patients [44], while others argue that mandating disclosure may present more harm [45,46]. Other relevant considerations include exploring whether healthcare professionals should disclose the value assumptions behind the recommendations made by an AI-based tool, or if basic knowledge of how AI makes recommendations ought to be a part of healthcare professionals’ medical training and/or continued education [47,48].

### 3.13. Account for the Risks of Automation or Technology Bias

Automation bias arises when healthcare professionals rely too heavily on AI tools and overlook conflicting information. In contrast, technology bias occurs when professionals dismiss AI recommendations in favor of their own judgment, often due to fear or distrust of new technology. Both biases can lead to improper use of AI tools. Different countries may also have varying perspectives on assigning credit or blame to AI, which can affect how these tools are used. Notably, attributing greater blame to AI may reduce automation bias [17,49]. Regulations should account for these biases as much as it is possible to promote the appropriate use of AI tools. Educating healthcare professionals about the potential risks may be a good starting point.

### 3.14. Clarify the Suitable Level of Critical Review by Professionals on AI Outputs to Avoid Deskilling

The use of AI tools should not result in the deskilling of professionals or the dehumanization of care. Healthcare professionals should remain actively involved, using AI to enhance their skills rather than as a replacement for their judgment. However, requiring them to review every AI-generated recommendation could undermine the efficiency benefits of AI. For example, one study showed that endoscopists’ behavior may be negatively affected by continuous exposure to AI-assisted colonoscopies, resulting in a reduction in the adenoma detection rate (ADR) of standard non-AI-assisted colonoscopies [50]. In an effort to combat such potential deskilling, the National University Health System (NUHS) in Singapore has implemented ‘AI-free periods’ where healthcare professionals refrain from the use of AI in the clinical setting. However, as pointed out by Adjunct Professor Ngiam Kee Yuan, head of NUHS’ Artificial Intelligence Office, it is not clear that AI will inevitably provoke deskilling [51].

Recommendations for a suitable level to avoid deskilling will depend on the skills involved [52]. It may be justifiable to make certain tasks fully automated, while others may need more human oversight. A clearer picture of exactly which types of skills belong in each camp may be more appropriate than a blanket “AI-free” period for all.

Although our recommendations are intended to be conceptually generalisable beyond Singapore, it is worth making explicit the practical considerations for implementing them. One factor affecting such practical considerations is the government’s role in the healthcare sector of different jurisdictions. In jurisdictions where the healthcare sector is highly state-led or centrally coordinated, governments can more readily standardize expectations (e.g., around procurement requirements, patient involvement processes, and lifecycle oversight), making system-wide implementation more straightforward. In jurisdictions where the healthcare sector is characterized by a roughly even mix of public and private players, implementation barriers are likely to arise from fragmentation and uneven capacity: how well the recommendations can be implemented may depend on whether each particular hospital or clinic has access to the governmental regulatory and training infrastructure and technical capacity to carry out the ethical oversight. In predominantly market-driven and deregulated systems, how AI will be used in healthcare will depend on healthcare provider and vendor practices, and one would expect much variation in real-world ethical performance across sites. A point-by-point discussion of feasibility and potential implementation barriers for each recommendation is provided in Appendix E.

## 4. Conclusions

Our analysis found that while Singapore’s regulatory documents are generally aligned with global standards, they lack integration, specificity on patient involvement, and clear guidance on key ethical challenges such as bias, explainability, human oversight, and data protection. Recommendations include emphasizing justifiability over explainability, publishing a registry of approved AI tools, and refining liability frameworks. Although Singapore has kept pace with global AI governance, gaps remain in harmonization and ethical depth. These gaps are not unique to Singapore. As mentioned above, our aim is to offer insights into one approach to identifying gaps in AI in healthcare guidance and regulation, especially in how they relate and integrate with other ethical and policy documents in healthcare. This process may be useful for other countries.

We note that one limitation of our paper is that we do not offer a more granular typology to help drive operational actionability of our recommendations. We recognize that risks may be separated by modality, autonomy level, or regulatory class and that different tool classes pose different implications for risk (i.e., locked vs. adaptive models, assistive vs. autonomous systems, and administrative vs. clinical tools). However, the aim of this paper is to address the wider ethical and philosophical issue driving each risk rather than offer a prescriptive driver of operational change in policy, though we do think our approach may result in such change nonetheless. Each recommendation offers a way to think about a problem that can then be applied to different contexts, where more technical arguments can follow. Though factors like priority, cost, implementation feasibility, and risk will vary by country and context, our aim is to offer other countries a model for identifying their own gaps.

We also note another limitation in that due to the wide range of recommendations produced, we are unable to provide a distinct feasibility analysis for each recommendation (though they are all based on existing good practice literature). Implementation feasibility will thus depend on the specific regulatory landscape of the nation adopting our strategy including their inter-institutional capacities, resources, and structural maturity. Further work within jurisdictional context would be needed for such implementation analysis. 

As AI technologies continue to be increasingly integrated into medical practice, there is an urgent need for more robust ethical and regulatory guidelines on the use of AI that address the issues discussed in this article. By aligning regulatory guidelines with these recommendations, Singapore will not only reap the benefits of appropriately integrating AI into the local healthcare landscape but also provide invaluable insights and lessons for other nations navigating similar challenges. Our recommendations offer a way to think about addressing these problems, which should result in enhanced trust, equity, and effectiveness in AI deployment in healthcare, and offer a model for international policy development. We note the ongoing evaluative nature of AI Ethics and seek to offer conceptual recommendations that keep pace with the fast-paced progress of these emerging technologies.

## Figures and Tables

**Table 1:** Savulescu et al.’s review of the nine main risks arising from the use of AI in medicine.

Risk	Description
Effectiveness, Reliability, and Evaluation	While pharmaceuticals require strict evaluation via clinical trials, this is not always feasible or necessary for the evaluation of AI tools. Instead, assessment is needed in the context in which the AI is actually being deployed, which introduces risks surrounding how humans will use the tool in practice.
Justice, Inequality, Bias, Discrimination, and Fairness	Bias in the social context interacts with algorithmic and statistical bias in AI, risking the exacerbation of health disparities.
Privacy and Confidentiality	AI systems require big data and risk breaches of privacy and confidentiality, raising concerns regarding consent, anonymization, and proper data management.
Machine Paternalism and Respect for Autonomy	AI may risk undermining patient autonomy by making decisions without considering patients’ values.
Accommodating Value Pluralism and Disagreement	Diversity of patient values and preferences requires accommodating differing views without imposing any one approach over another.
Responsibility	In the event of errors or harm, ensuring accountability among developers, clinicians, and healthcare institutions is complex.
Trust	A relationship of trust should be upheld among all stakeholders in the deployment of an AI tool such that each party can rely on and feel confident in the system’s ethical use.
Need for Explanation and Justification	Explainable AI ensures clinicians and patients understand how decisions are made, fostering trust and informed consent, but explainability may not always be necessary. Instead, justification of AI decisions better serves to uphold patient well-being and autonomy.
Obsolescence, Dehumanization, and Deskilling	AI systems should seek to keep a level of human oversight such that there is not a loss of human interaction in care. Healthcare professionals should remain skilled in their craft and not be over-reliant on new technologies.

## Data Availability

No new data were created or analyzed in this study. Data sharing is not applicable to this article.

## Appendix A. Definitions for Key Stakeholder Groups

We define the three key groups that form the focus of our paper.

(i) Service Provider: Person(s) within an organization responsible for selecting healthcare AI solutions for clinical/ops use, or implementing use of a healthcare AI solution in a healthcare setting. e.g., person on a committee responsible for the selection and procurement of an AI solution for a hospital, or an “AI champion” responsible for education on AI and encouraging AI use.

(ii) Developer: Person(s) creating a healthcare AI solution, adapting a foundation model, etc., for use in clinical/operations workflow within a healthcare setting. This could include clinicians within a healthcare setting if the clinicians take on a developer role in developing AI solutions intended for use in their own clinical workflows.

(iii) Healthcare Professionals and Patients: Doctor, dentist, nurse, or allied health professional providing AI-augmented patient care, or any individual patient (whether or not he or she is undergoing any medical treatment, care, or therapy). As per MOH’s recommendation, we combine the “healthcare professionals” and “patients” groups. MOH’s rationale for this is due to the fact that the ethical concerns and considerations of both groups are likely to be deeply intertwined.

## Appendix B

Common Ethical Themes 1. Fairness 2. Responsibility 3. Transparency 4. Explainability 5. Building trust and confidence 6. Patient-centricity 7. Reduce bias (Continuous learning) 8. Confidentiality and privacy in Cybersecurity 9. Risk Management: Balancing safety and performance. 10. Ethical oversight to prevent harm.	Common Gaps 1. Lack of direct guidance for patients 2. Insufficient patient engagement/involvement 3. The challenge of ethical proportionality 4. Trust versus reliability 5. Addressing automation and technology bias 6. Level of appropriate human oversight 7. Addressing machine paternalism 8. Accommodating value pluralism 9. Defining bias and describing nuances 10. Examining trust versus reliability 11. Justifiability versus explainability

## Appendix C. List of 9 Documents

1. HSA Regulatory Guidelines for Software Medical Devices—A Life Cycle Approach. This document provides guidance on managing software medical devices throughout their life cycle, from design and development to post-market surveillance. The guidelines emphasize the importance of ensuring the safety, performance, and cybersecurity of software medical devices, including AI-based Medical Devices (AI-MDs). It is relevant to stakeholders who are involved in software medical device development and/or supplying such devices in Singapore, mainly developers and implementers.
2. MOH Artificial Intelligence in Healthcare Guidelines (AIHGle). AIHGle accompanies the HSA Health Products Act and its subsidiary legislation and guidance documents. It provides a set of good practices for developers and implementers involved with AI in healthcare. The primary aim is to improve clinical and public trust in AI by encouraging the safe development and implementation of primarily AI-MDs and other AI applications. It is relevant to developers and implementers.
3. Regulatory Guidelines for Telehealth Products. This document provides regulatory guidance for telehealth products classified as medical devices, including mobile apps. It follows two approaches: risk-based regulation and confidence-based regulation to ensure safe and efficient delivery of the products. It is relevant to developers and implementers.
4. ACE Overview for New and Emerging Technologies. This document is a rapid overview by the ACE (Agency for Care Effectiveness), summarizing the current state, clinical applications, regulatory considerations, and implementation challenges of AI in healthcare, specifically within the context of Singapore. This document only comments on the above topics and cannot in itself be used to guide or regulate professionals. It does, however, point out gaps in existing regulations and guidelines on ethical considerations related to the use of AI in healthcare. Thus, this document informs the identification of such gaps in our analysis.
5. SMC Ethical Code and Ethical Guidelines (2002 and 2016 editions) and Handbook on Medical Ethics (2016 edition). Developed by the Singapore Medical Council, this set of documents presents the fundamental tenets of medical ethics that inform the standards of professional conduct for doctors in Singapore. It is relevant for Individuals (healthcare professionals).
6. SDC Ethical Code and Ethical Guidelines (updated 2019). This document captures the set of ethical codes and guidelines for dentists. Relevant to the Individuals (healthcare professionals) group.
7. Allied Healthcare Professionals Code of Professional Conduct. This document captures the set of ethical codes and guidelines for allied healthcare professionals in Singapore. It is relevant to the Individuals (healthcare professionals) group.
8. SPC Code of Ethics. This document captures the set of ethical codes and guidelines for pharmacists in Singapore. It is relevant to the Individuals (healthcare professionals) group.
9. Nurses and Midwives Code. This document captures the set of ethical codes and guidelines for nurses and midwives in Singapore. It is relevant to the Individuals (healthcare professionals) group.

## Appendix D

### International Comparison of Key Regulatory AI Frameworks

**Jurisdiction**	**Key Regulatory Frameworks**	**Ethical Considerations**
USA	Food and Drug Administration (FDA) oversees AI/ML-based medical devices under the Software as a Medical Device (SaMD) framework. The 2021 Action Plan includes a lifecycle approach. Other guiding documents include the *Blueprint for Trustworthy AI* and *Good Machine Learning Practice*. The *AI Bill of Rights* also explains the roles and rights of the public, including patients.	Fairness, transparency, and managing risks of bias in AI development. Trustworthiness and explainability emphasized.
UK	SaMD is regulated by the Medicines and Healthcare products Regulatory Agency (MHRA). The *Software and AI as a Medical Device Change Program* in 2021 addresses post-market evaluation. The MHRA roadmap in 2022 also covers manufacturer vigilance.	Focus on patient safety, reducing bias, explainability, and ensuring transparency throughout the AI lifecycle.
EU	EU AI Act (2024) [8] introduced a risk-based classification with stringent regulations for high-risk systems, such as AI in healthcare, requiring conformity assessments, human oversight, and transparency. It also mandates registration of high-risk systems for public transparency, and sets strict rules for general purpose AI.	Emphasize fairness, non-discrimination, and privacy protection. Strong focus on transparency, human oversight, and bias reduction in healthcare applications.
Australia	The Therapeutic Goods Administration regulates SaMDs with a risk-based approach. *Safe and Responsible AI in Healthcare Legislation and Regulation Review* was launched in September 2024 for this purpose.	Emphasize minimizing bias, ensuring reliability, and maintaining safety in AI healthcare applications.
China	The National Medical Products Administration oversees AI with a focus on lifecycle management, cybersecurity, and risk factors. There are stricter regulations for AI in healthcare.	Concerns around transparency, data security, managing algorithmic bias, and ensuring explainability.
Brazil	Draft AI Law classifies healthcare AI as high-risk, requiring impact assessments and strict liability for AI damages.	Ethical considerations include accountability, transparency, and ensuring patient safety in high-risk systems.
Japan	SaMD are regulated by *Pharmaceuticals and Medical Devices Act*, *Guidelines on the Applicability of Programs as Medical Devices*, *Digital Transformation Action Strategies in Healthcare for SaMD*, and *Next Generation Medical Infrastructure Act*.	Focus on safety, accountability, reducing bias, ensuring trust and data privacy while keeping up with the updates of the devices.
Singapore	AI is regulated under the SaMD framework. The AIHGle guidelines emphasize good practices for developers and implementers, including quality management. Various other documents, such as the PDPA, may have implications for AI in healthcare but do not directly address AI in healthcare.	Focus on patient-centricity, transparency, reducing bias, and ensuring trust and accountability.

## Appendix E

**Table A1:** Recommendations distinguished by priority, cost, implementation feasibility, who has authority, and risk of legal incompatibility (Note that we did not include the recommendation to “Account for the risks of automation or technology bias” because it is not a very specific policy suggestion so it is not feasible to assess its cost/feasibility etc in a very meaningful way).

Recommendation	Priority	Cost	Implementation Feasibility	Who Has Authority	Risk of Legal Incompatibility	Note on Implementation Beyond Singapore
Involve patient perspectives in the development and implementation of AI in health	High	Medium	Variable: feasible for developers with established Voice of the Customer teams, and for projects backed by the government with established public engagement avenues, less so for those without.	Developers and implementers may take charge; regulators may set up guidelines (though not hard laws) to incentivize patient-centric AI.	Low	Regulators may account for the presence/absence and quality of public/patient engagement when assessing AI medical device or software for approval. However, jurisdictions may adopt a laissez-faire approach to AI at their own discretion (this holds for all subsequent recommendations).
Indicate that AI-based tools may be evaluated based on their justifiability if they are not explainable	Low	Low	High	Regulators	Low	Regulators may include statements to such effect in their AI policy documents.
Developers and implementers should, depending on the context of use, warn users against the tendency to anthropomorphize AI-based tools	Medium	Low	High	Developers and implementers	Low	Regulators may include statements to such effect in their AI policy documents.
Provide guiding tools for ethical analysis of proportionality in data collection and making ethical trade-offs in general	Medium	Low	Variable: users need training to conduct ethical analysis effectively. This is feasible for states with established ethics training infrastructure for medical and allied health professionals like Singapore, but less so for those without.	The state needs to standardize such tools if centralized ethics training is provided; otherwise, implementers like hospitals and clinics may take charge	Low	Regulators in low-resource settings may tolerate some inconsistency in ethical capacity across medical institutions by, for instance, requiring ethical accreditation for higher-tier hospitals.
Publish approved AI/ML enabled devices for medical use	Low	Low	Variable: feasible for regulators with close oversight of medical devices, less so for the more laissez-faire regulators.	Regulators	Low	-
Extend personal data protection beyond identified data	High	Low	Variable: data custodians and ethics review board members need training to be informed about the risk of re-identification with AI tools. This is feasible for states with established clinical and research ethics training infrastructure, but less so for those without.	Regulators	Low: privacy laws should have already accounted for potential technological advancements (e.g., the EU GDPR Recital 26 posits that assessment of data identifiability should account for “all the means reasonably likely to be used”, which should include the latest AI tools).	-
Clarify the distribution of liability between healthcare professionals, implementers and developers	High	Low	High	Regulators	Medium: existing laws may already lay out rules for liability attribution; regulators must avoid clashing with those laws.	Regulators may include statements to such effect in their AI policy documents and/or doctor/nurse/dentist ethical codes.
Clarify how implementers should deliberate the appropriate amount of human oversight of AI appropriate by weighing risks and benefits of full automation	Medium	Low	High	Regulators	Low	Regulators may include statements to such effect in their AI policy documents.
Require calibration of tools to be culturally sensitive	High	Medium	Variable: feasibility may be constrained by the (un)availability of relevant training data.	Developers and implementers may take charge; regulators may set up guidelines (though not hard laws) to incentivize culturally sensitive AI.	Low	Regulators may include statements to such effect in their AI policy documents.
Clarify when and how, if ever, professionals may still use a biased AI	Low	Low	High	Regulators	Low	Regulators may include statements to such effect in their AI policy documents and/or doctor/nurse/dentist ethical codes.
Clarify the scope of patient rights in relation to the use of AI-based tools	High	Low	High	Regulators	Low	Regulators may include statements to such effect in their AI policy documents.
Guiding documents regarding the doctor-patient relationship on their rights and/or duties with respect to AI use in healthcare	High	Low	High	Developers and implementers may take charge; regulators may make recommendations (though not hard laws).	Low	Regulators may include statements to such effect in their AI policy documents and/or doctor/nurse/dentist ethical codes.
Clarify the suitable level of critical review by professionals on AI outputs to avoid deskilling	High	Low	High	Developers and implementers may take charge; regulators may make recommendations (though not hard laws).	Low	Regulators may include statements to such effect in their AI policy documents and/or doctor/nurse/dentist ethical codes.

## Appendix F

### Case Study 1: AI for Screening and Referrals for Diabetic Retinopathy (DLSDR)

This case explores the implementation of an AI tool used to screen and refer patients for Diabetic Retinopathy (DLSDR) in Singapore. The AI system was shown to refer Malays to specialists less often than other groups, creating a disparity in diagnosis. Despite this, overall referrals for Malays increased, improving early detection for this group. The ethical challenge is balancing utility and equity: although the AI tool reduced underdiagnosis across all groups, it also potentially widened the disparity between Malays and non-Malays. The ethical argument here is that it is sometimes justifiable to implement a biased AI tool if it reduces existing biases and offers significant utility gains, even if it worsens some inequalities.

Key Ethical Argument: It is ethically justifiable to deploy biased AI if it improves outcomes across groups and reduces some forms of bias, even if it does not eliminate all disparities.
